# Systematic Review and Meta-Analysis of the Financial Impact of 30-Day Readmissions for Selected Medical Conditions: A Focus on Hospital Quality Performance

**DOI:** 10.3390/healthcare12070750

**Published:** 2024-03-29

**Authors:** Iwimbong Kum Ghabowen, Josue Patien Epane, Jay J. Shen, Xan Goodman, Zo Ramamonjiarivelo, Ferhat Devrim Zengul

**Affiliations:** 1 Department of Healthcare Administration, School of Public Health, University of Nevada Las Vegas, Las Vegas Nevada, NV 89154, USA; iwimbong@unlv.nevada.edu (I.K.G.); jay.shen@unlv.edu (J.J.S.); 2 Department of Healthcare Administration, School of Public Health, Loma Linda University, Loma Linda, CA 92354, USA; jepane@llu.edu; 3 University Libraries, School of Public Health, University of Nevada Las Vegas, Las Vegas Nevada, NV 89154, USA; xan.goodman@unlv.edu; 4 School of Health Administration, College of Health Professions, Texas State University, San Marcos, TX 78666, USA; zhr3@txstate.edu; 5 Department of Health Services Administration, School of Health Professions, University of Alabama Birmingham, Birmingham, AL 35294, USA

**Keywords:** 30-day readmission, hospitals, cost, meta-analysis, quality improvement, financial performance

## Abstract

Background: The Patient Protection and Affordable Care Act (ACA) established the Hospital Quality Initiative in 2010 to enhance patient safety, reduce hospital readmissions, improve quality, and minimize healthcare costs. In response, this study aims to systematically review the literature and conduct a meta-analysis to estimate the average cost of procedure-specific 30-day risk-standardized unplanned readmissions for Acute Myocardial Infarction (AMI), Heart Failure (HF), Pneumonia, Coronary Artery Bypass Graft (CABG), and Total Hip Arthroplasty and/or Total Knee Arthroplasty (THA/TKA). Methods: Eligibility Criteria: This study included English language original research papers from the USA, encompassing various study designs. Exclusion criteria comprise studies lacking empirical evidence on hospital financial performance. Information Sources: A comprehensive search using relevant keywords was conducted across databases from January 1990 to December 2019 (updated in March 2021), covering peer-reviewed articles and gray literature. Risk of Bias: Bias in the included studies was assessed considering study design, adjustment for confounding factors, and potential effect modifiers. Synthesis of Results: The review adhered to PRISMA guidelines. Employing Monte Carlo simulations, a meta-analysis was conducted with 100,000 simulated samples. Results indicated mean 30-day readmission costs: USD 16,037.08 (95% CI, USD 15,196.01–16,870.06) overall, USD 6852.97 (95% CI, USD 6684.44–7021.08) for AMI, USD 9817.42 (95% CI, USD 9575.82–10,060.43) for HF, and USD 21,346.50 (95% CI, USD 20,818.14–21,871.85) for THA/TKA. Discussion: Despite the financial challenges that hospitals face due to the ACA and the Hospital Readmissions Reduction Program, this meta-analysis contributes valuable insights into the consistent cost trends associated with 30-day readmissions. Conclusions: This systematic review and meta-analysis provide comprehensive insights into the financial implications of 30-day readmissions for specific medical conditions, enhancing our understanding of the nexus between healthcare quality and financial performance.

## 1. Introduction

The Patient Protection and Affordable Care Act of 2010, which is a federal law, has the primary purposes of expanding the health insurance coverage of US citizens and improving the quality of healthcare delivery. However, it presents financial threats and opportunities to many hospitals across the USA [1]. The enactment of the Patient Protection and Affordable Care Act (ACA) led to the implementation of initiatives such as the Hospital Readmissions Reduction Program (HRRP) to enhance healthcare quality through reducing hospital readmissions. Hospitals reimbursed under the Inpatient Prospective Payment System (IPPS) are expected to provide more and more predefined quality performance indicators. These changes in how hospitals are reimbursed can cause some hospitals to undergo financial sanctions [1,2]. The widespread expressions of chronic illnesses in the baby boomer generation translate into increasing demand for medical services. Hospitals face financial strains as the demand for quality services increases with higher patient expectations, coupled with penalties from the Centers for Medicaid & Medicare Services (CMS) Hospital Readmissions Reduction Program (HRRP) initiatives [3,4]. Historically, through the HRRP program, the CMS can withhold up to 3% of reimbursements for readmissions within 30 days, which exceeds national standards. The use of national standards is being criticized for not accommodating the case mix of patients [5,6]. Thus, active 2019 hospitals are ranked in quintiles depending on the proportion of dual-eligible patients that each hospital serves. Therefore, the new methodology will compare each hospital to the median readmission rates of its cohorts. It is unknown how this new methodology will affect the cost of readmission. 

### 1.1. New Contribution 

Despite the increasing dependence on 30-day readmission rates and patients’ case mix in determining hospital reimbursements, there seems to be limited research on how payment based on 30-day readmission is related to the cost of readmissions. This lack of attention is evident because no meta-analysis on the relationship between financial performance and 30-day readmission rates has been published. To our knowledge, this is the only meta-analysis of the literature to date exploring the relationship between 30-day readmission rates and costs. Given that meta-analysis is considered the gold standard in analyzing, synthesizing, and integrating available literature on quality and financial outcomes [7], this study has significant potential for informing future research. A synthesis of the novel literature is included in this study to account for recent trends on how hospitals perform financially on the indicators of 30-day readmission rates, as this is needed to streamline our meta-analysis and guide future studies.

This study aims to contribute to the literature by adding more knowledge on previous studies [8,9] by specifically looking at studies including the quality variables of 30-day readmission rates and financial performance, which were not included in previous studies. A focus on this neglected quality aspect will allow for more inferences to be drawn about salient variables in hospital settings implementing the HRRP.

### 1.2. Conceptual Framework

This study adopted the quality–cost framework using the Donabedian Model of structure, process, and outcomes [8,9,10,11,12]. Our quality measure is the reduction in the cost of 30-day readmission rates. The structure represents the resources that hospitals use to reduce 30-day readmissions, like increasing staff ratios, equipment, and institutional/treatment protocol [13,14]. The structure also encompasses the formal and informal systems through which healthcare is financed, like the insurance structure, healthcare policies, healthcare worker availability, and available healthcare delivery systems [15,16] Within the conceptual framework’s process dimension, we delve into the dynamic elements of patient care: assessments, examinations, and a spectrum of healthcare interactions. This expansive perspective recognizes that the efficacy of these processes is intricately linked to the overarching cost dynamics of readmissions. Our study underscores the critical role that various processes play in influencing healthcare expenditure, emphasizing the need to scrutinize and optimize these aspects to mitigate the costs associated with 30-day readmissions. By unpacking the multifaceted process dimension, our research aims to provide a robust understanding of how healthcare processes contribute to the overall financial landscape in the context of readmissions [15]. This comprehensive perspective recognizes that the effectiveness of these processes is intricately linked to the overarching cost dynamics of 30-day readmissions [16].

We carried out a literature review of how hospital readmissions affect financial performance and located the readmission cost as our variable of interest for the meta-analysis rooted in the rationale provided by the quality–cost framework. This framework delineates the relationship between the quality aspects of the cost of readmission in hospitals. It maps the structure and determines the process, ultimately leading to outcomes. For the structure, we consider the quality improvement measures for the subdomains of quality-specific diseases of 30-day readmission for Acute Myocardial Infarction (AMI), Chronic Obstructive Pulmonary Disease (COPD), Heart Failure (HF), Pneumonia, Coronary Artery Bypass Graft (CABG) surgery, Elective Primary Total Hip Arthroplasty and/or Total Knee Arthroplasty (THA/TKA), as well as quality improvement measures [17].

**Figure 1:**
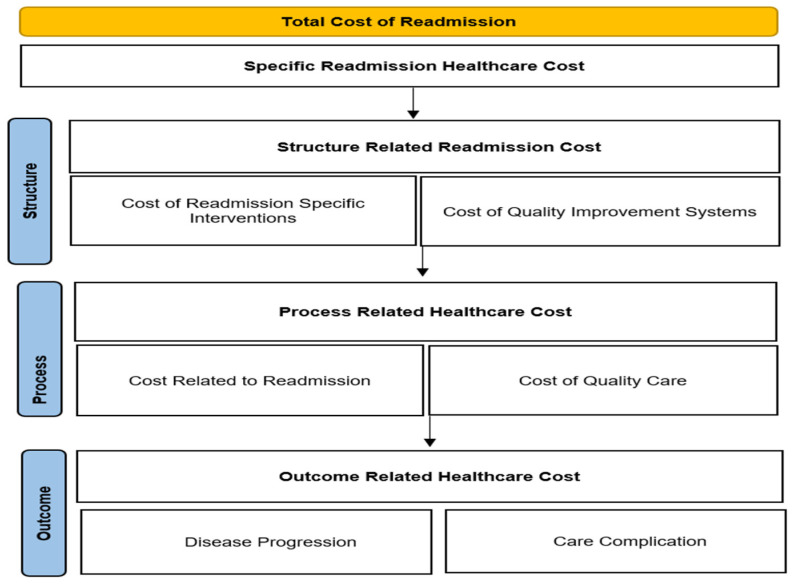
Cost that can be influenced by quality components of readmission. Adopted from [12].

Within this framework (Figure 1), we delineate the structure to include quality improvement measures to reduce readmissions for AMI, COPD, HF, Pneumonia, CABG Surgery, and THA/TKA. The process includes subdomains related to medical errors and appropriate care that affects readmissions, while outcomes involve disease progression and care complications for various medical conditions. Considering that each of these quality attributes inherently has cost implications for monitoring and evaluation and subsequently influences healthcare costs, the conceptual foundation is built upon the foundational work of previous studies [18,19] which identified the dimensions of profitability: profitability, liquidity, capital structure, activity, cost, revenue, and utilization. Conducting a thorough systematic review that reveals other financial performance measures was crucial to understand the research landscape in this domain and to justify why cost was isolated for the meta-analysis. We derived the following research question building on this framework. 

What is the relationship between financial performance variables reported as independent factors for 30-day readmission, the different pathological conditions associated with 30-readmission, and the significant findings derived from the average cost of readmission?

Meta-analysis precedes the literature review to comprehensively understand the interplay between financial aspects and readmission. This approach enables us to glean insights into the intricate relationship between quality improvement, financial performance measures, and the cost measure of financial performance with hospital readmission. 

## 2. Methods 

**Figure 2:**
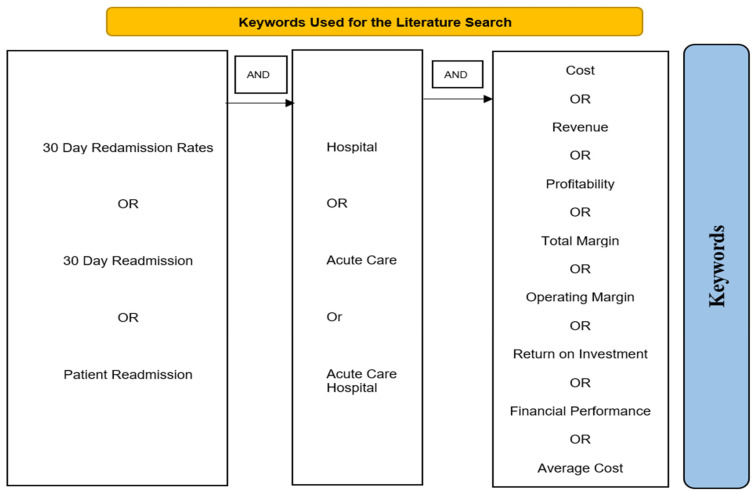
Keywords used for the literature search.

We carried out a meta-analysis on the Covidence software following the Preferred Reporting Items for Systematic Review and Meta-Analysis (PRISMA). A three-step procedure was used for the review. The keywords for financial performance, 30-day readmission rates, and the hospital setting, as defined by the Boolean operators OR/AND, were used to arrange the keywords (Figure 2). The search strategy used quality, financial performance headings and keywords and their combinations “30-day readmission rates”, “patient readmission”, “re-hospitalization”, “reoperation”, AND/OR “hospital” “acute care”, “ acute care hospital” “critical care” AND/OR “cost”, “revenue”, “profitability”, “total margin”, “operating margin”, “return on investment”, “financial performance”, “financial”, “accounting”, “financing”, “activity”, and “outcome.” Peer-reviewed articles were located using the following databases: ABI/INFORM, Web of Science, Scopus, PubMed/MEDLINE Medline, Embase, and Academic Search Premier.

**Figure 3:**
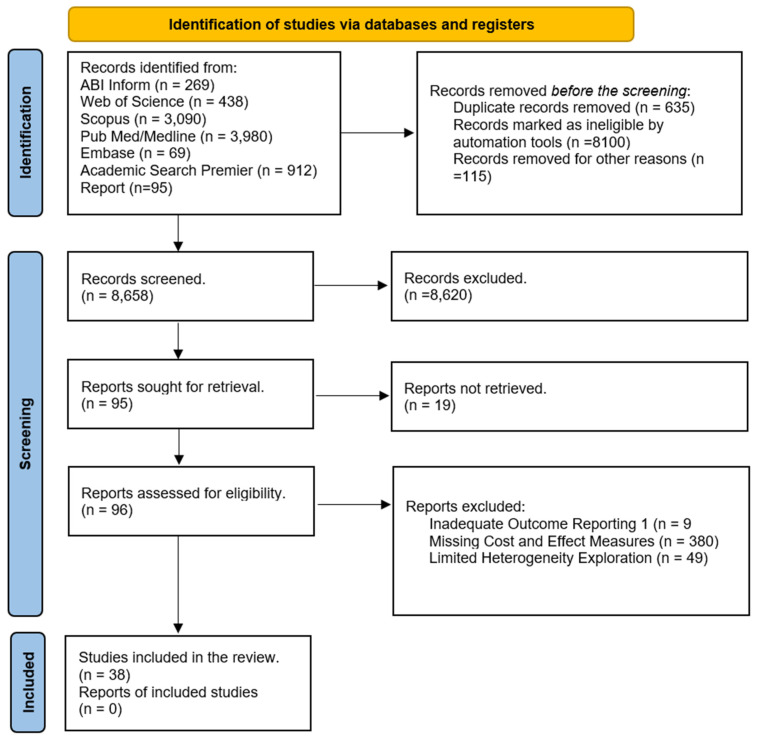
Cohort selection for systematic review and meta-analysis.

This meta-analysis used PRISMA guidelines (Figure 3). We considered the following types of studies for inclusion: full original research papers written in the English language, randomized or non-randomized controlled trials, prospective or retrospective cohort studies, cross-sectional studies, pilot studies, and studies from the USA. For optimal search outcomes, we extracted peer-reviewed articles, gray literature written only in English, published between January 1990 and December 2019, and updated in March 2021 and February 2024. The effects described were proven change or no change in financial performance for 30 days of readmission. We excluded the studies that did not include the hospital financial performance as the outcome or comparator and qualitative studies without empirical evidence for hospital financial performance. Abstracts were screened for studies not carried out within the United States to account for variations in international epidemiologic, economic, and medical practice. 

### 2.1. Outcomes

We extracted the independent variables of 30-day readmission rates as the dependent variable. We calculated the inflation-adjusted financial performance using the standardized abstraction protocol and the Covidence tool by three abstractors. Financial performance outcomes included cash flow margin, charges, income, cost revenue, operating margin, return on investment, operating expenses, operating revenue, return on assets, total margin, operating expense, and one-year subsequent Medicare spending. We analyzed studies conducted with the patient populations of hospitals and hospital populations separately to reduce any bias related to hospitals’ financial performance. A separate analysis was conducted for all-cause readmission rates, acute myocadiac infarction, heart failures, and other illnesses. The studies were analyzed for design and adjustment for confounding factors for possible effect modifiers. Various authors have used different units of measurement for financial performance. We adapted to use articles reporting financial performance as a measure of the USD value and eliminated articles that only reported quality aspects.

We took a second secondary review of abstracted publications from Covidence to determine whether articles on the borderline met the inclusion criteria. This process illuminated 11 articles primarily linked to the methods of reporting economic outcomes. We identified 24 studies estimating the attributable cost of all-cause 30-day readmission. Within the included articles, minimal variations in methods, data sources, and settings could not be avoided. All included articles generated average attributable costs from readmission. In cases where other variables, in addition to cost, were reported, we only considered the cost and charge component. Using the charge, we estimated the cost using a cost-to-charge ratio of 0.50, as used by [1,2].

To bolster the methodological rigor and align more closely with PRISMA guidelines, studies that reported the effect measures for each outcome were explicitly stated, specifying the metric employed, be it risk ratio, mean difference, or other relevant measures. In addressing heterogeneity, our discussions elucidate the methods applied, such as subgroup analyses or meta-regression, to explore potential variations among the study results comprehensively. To tackle the reporting bias, we employed Monte Carlo simulations to assess and mitigate the biases arising from missing results. These refinements enhance the transparency and thoroughness of the meta-analysis, fostering a more robust adherence to PRISMA guidelines and affording readers a nuanced insight into the methodologies underpinning this systematic review. Our study sought to estimate the mean cost of 30-day readmissions, a probabilistic outcome subject to uncertainty due to various factors such as patient characteristics, treatment effectiveness, and healthcare processes. Monte Carlo simulation is well suited for handling such uncertainty by repeatedly sampling from input parameter distributions to estimate the distribution of possible outcomes. In this case, the simulation would allow for the assessment of the uncertainty around the mean cost estimates and provide confidence intervals.

### 2.2. Statistical Analysis

The results reported in the articles were very heterogeneous. For example, different financial performances were reported (Marginal Cost, Incremental Cost, Operating Revenues, Operating Expenses, and Operating Margin). Operating Margin, Cost, and Return on Investment) with a specific lens on readmission costs. The reporting of cost also differed across papers as some reported raw USD values while others reported mean/median costs. In addition, different pathological conditions were reported. Most of the studies did not mention controls (ideally 30-day unadjusted mortality rates), making it exceedingly difficult to perform a meta-analysis.

The literature review results provided average estimates of the cost of a 30-day readmission. For each study included, a weighted average of the point estimate was used to calculate the cost estimate relative to the sample size. To assess the consistency of the association between 30-day readmission rates and financial performance outcomes across several studies, we conducted a Monte Carlo simulation to develop confidence intervals (CI) for every point estimate. We achieved this by generating our data to see the trend and creating an estimator to see how close we are to the trend. We analyzed the studies separately, considering the data years to adjust for inflation. Considering that our data came from various sources, we chose Monte Carlo simulations because of their ability to realistically estimate uncertainty.

Meta-analysis was chosen as the primary research method to synthesize data from multiple studies and provide a robust estimation of the mean cost of 30-day readmissions across various medical conditions. This method allows for the integration of findings from disparate studies to derive more precise and generalizable conclusions.

**Figure 4:**
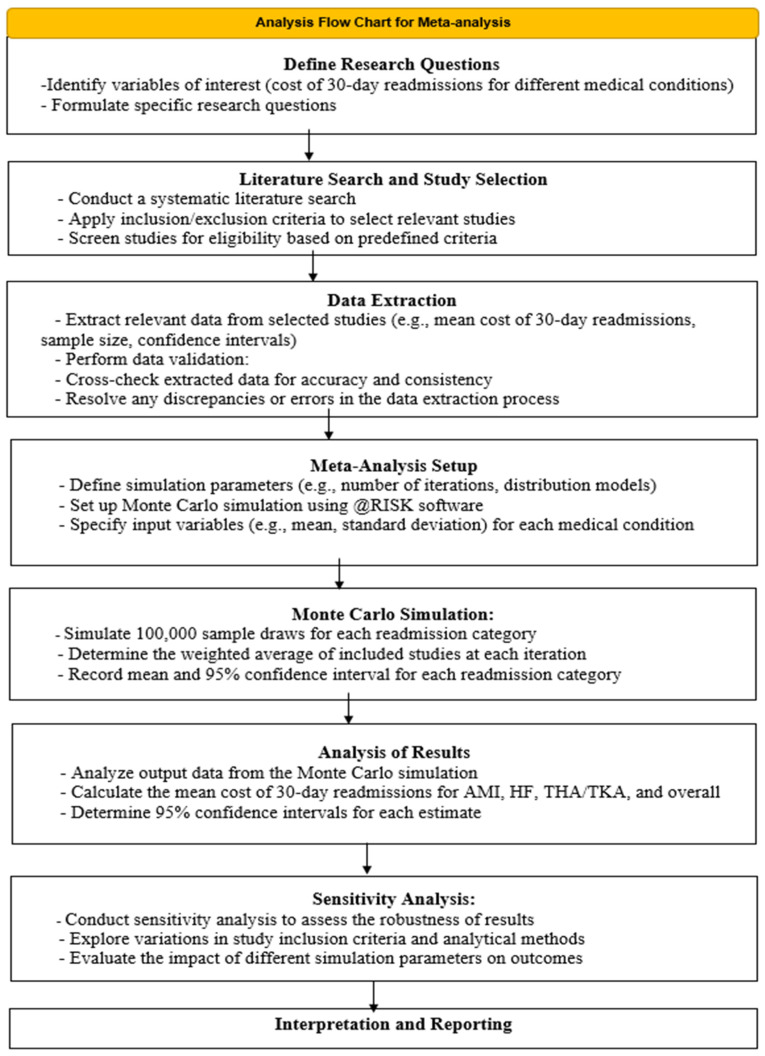
Analysis flow chart for meta-analysis.

The meta-analysis process involved several key steps to ensure methodological rigor and validity using the meta-analysis flow chart depicted in Figure 4. 

To develop the confidence interval through Monte Carlo simulations, we conducted a series of sensitivity analyses using the variant approach suggested in the research [20,21] for health service research cost estimates. For each study’s probabilistic distribution of a cost estimate, a Monte Carlo simulation was conducted with 100,000 trials. The output was a triangular and general distribution with a low end, most probable point estimate, and a high end.

## 3. Results 

The articles from the literature search were carefully reviewed based on inclusion/exclusion criteria. A total of 38 studies were found in the systematic review to be eligible for inclusion and were considered for further analysis (Table A1). Studies were further categorized into five different categories (Acute Myocardial Infarction (AMI), Chronic Obstructive Pulmonary Disease (COPD), Heart Failure (HF), Pneumonia, Coronary Artery Bypass Graft (CABG) surgery, and Elective Primary Total Hip Arthroplasty and/or Total Knee Arthroplasty (THA/TKA)) based on the disease condition. 

A Monte Carlo simulation method was carried out to estimate the average cost of 30-day readmission conditions due to its capacity to provide reasonably accurate uncertainty forecasts. For the Monte Carlo analysis, first, we created the confidence intervals for the 30-day readmission cost reported in this article. Then, the identified 38 studies providing the reasonable cost estimates of the attributable cost of readmissions (Figure 4). We identified 4 studies for AMI, 6 studies for HF, 6 studies for THA/TKA, and 22 studies for all other readmissions. 

We simulated the distribution for each suitable analysis before pooling the results and weighting the results by sample size. We followed the method described another meta-analysis simulating cost [21,22], which is based on three observations: point figures for the three experiments that make up the strongest indicator of central inclination, as well as lower and upper limits. For each readmission reported in the study, we used the 95 percent CI to set the endpoints for the distribution if it were either stated in the article or could be estimated so that 2.5 percent of the distribution falls below the lower and above the upper value. We then tested to see whether the modeled triangular distribution accurately matched the study results by setting the most possible value of the triangular distribution equal to the reported central propensity scale. 

**Figure 5:**
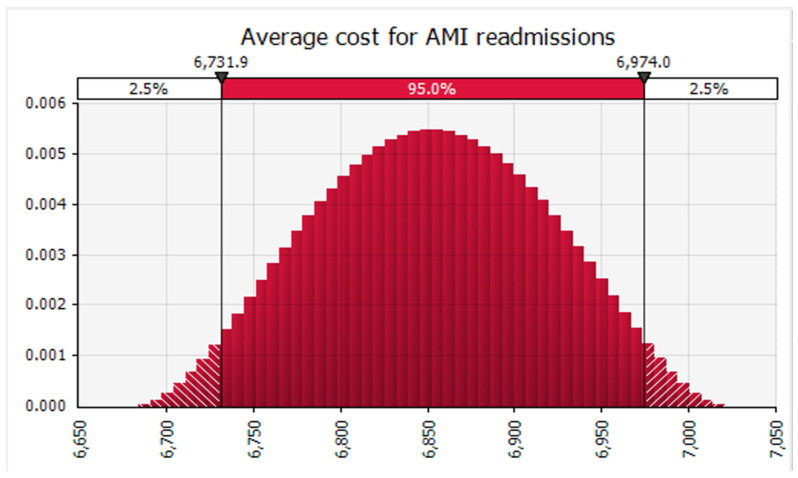
Average cost of 30 Day Readmission for Acute Myocardial Infarction.

**Figure 6:**
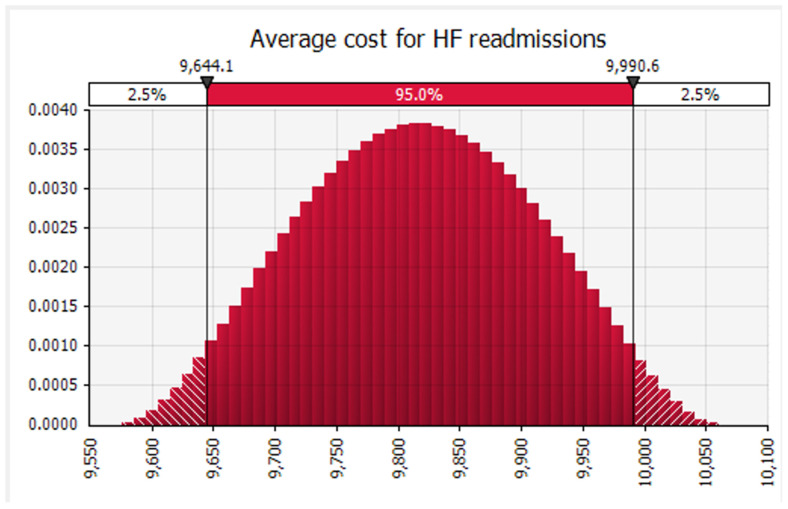
Average cost of 30 Day Readmission for Heart Failure.

**Figure 7:**
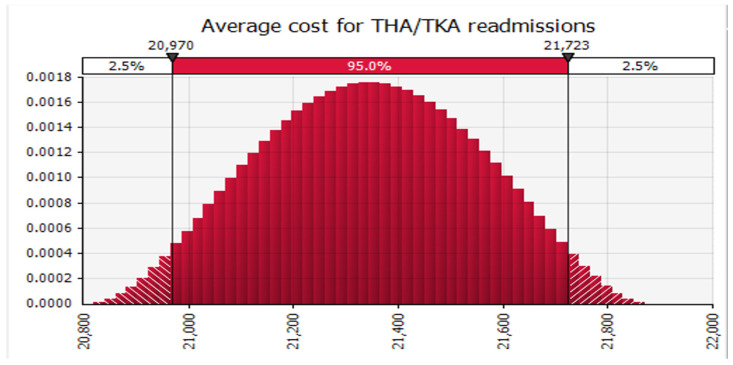
Average Cost of 30 Day Readmission for Total Hip Arthroplasty (THA) and Total Knee Arthroplasty (TKA) procedures.

**Figure 8:**
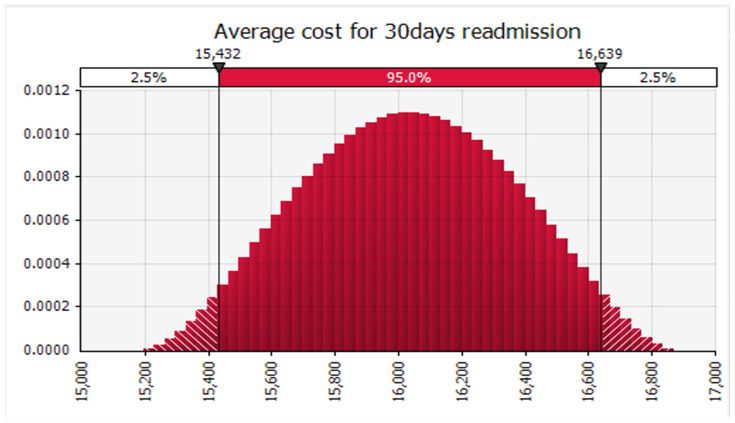
Average cost of 30 Day readmission.

Finally, we conducted Monte Carlo simulations using @RISK software to analyze the data. Specifically, we simultaneously simulated 100,000 sample draws from the modeled distribution for each related analysis across all readmission categories. At each iteration, we determined the weighted average of the included studies. We recorded the mean and 95 percent confidence interval obtained from the distribution of those 100,000 weighted averages for each readmission category. Subsequently, we calculated the mean cost of 30-day readmission for all conditions and specific conditions, such as AMI (Figure 5), HF (Figure 6), and THA/TKA (Figure 7), along with their respective confidence intervals. Monte Carlo simulations were carried out with the help of the Monte Carlo simulation software @RISK, version 7.6.1. (Palisade Corp., Ithaca, NY, USA). Following the Monte Carlo interactions seen in (Figure 8), the mean cost of 30 days readmission for all conditions is simulated at USD 16,037.08 (95% CI, USD 15,196.01–16,870.06). The mean cost of 30-day readmission for AMI is USD 6852.97 (95% CI, USD 6684.44–7021.08). The mean cost of 30-day readmission for HF is estimated at USD 9817.42 (95% CI, USD 9575.82–10,060.43). The mean cost of 30 days readmission for THA/TKA is simulated at USD 21,346.50 (95% CI, USD 20,818.14–21,871.85)”.

## 4. Discussion

Two decades after the landmark article “To Err is Human” by the Institute of Medicine (IOM), patient safety and quality improvements have been claimed to be at the forefront of many initiatives. The CMS implemented the readmission reduction program to reduce 30-day readmissions and improve quality-of-care efforts that can lead to significant cost reduction [23]. In this study, we conducted a systematic review and meta-analyses to explore the financial implications of a 30-day readmission reduction program. To achieve our goal, for the six pre-standardized unplanned readmission measures, following our search criteria, we found articles for AMI, HF, and THA/TKA that reported the attributable cost of readmission. We did not include readmissions for COPD, pneumonia, and CABG, as these conditions were not included in the 38 articles retained for this meta-analysis. These results were unexpected as most research has been performed on AMI, HF, and pneumonia [24,25]. This is true because the HRRP was established in 2010 with the initial target indicators AMI, HF, and pneumonia [2,26]. It is, therefore, surprising that the studies we found reposrted limited the attributable costs of pneumonia that meet our inclusion criteria.

In this section, we seek to bolster the robustness and credibility of our Monte Carlo simulation estimates by aligning them with real-world data from the Healthcare Cost and Utilization Project (HCUP) for the year 2018. This comparative analysis serves as a pivotal step in validating our findings. By referencing the HCUP data, we corroborate the accuracy of our Monte Carlo simulations, enhancing their overall credibility and robustness. This approach validates our estimates and significantly contributes to our comprehension of hospital readmissions, their associated costs, and their far-reaching impacts on healthcare quality and financial performance.

Our study estimated the average cost of 30-day all-cause adult hospital readmissions at USD 16,037.08. This estimate closely aligns with the data reported in HCUP, which indicated an average readmission cost of USD 15,200 for the same period. This consistency strengthens the validity of our estimates (HCUP, 2018). The average cost of readmission that we found through the meta-analysis was USD 16,870.06 (95% CI, USD 15,196.01–16,870.06). This amount is above the readmission rates reported by [27,28] for rural community hospitals at USD 2683 and USD 2248.21, respectively. On the other hand, the subacute hospitals had a significantly higher cost of readmission USD 15,563 [27]. These results indicate the geographical differences in the cost of readmission to build sustainable healthcare systems with unwarranted variety in the quality of care and cost. These challenges should be avoided. It is crucial to understand the geographical distribution of unplanned readmissions and how these variations impact the cost of readmissions [28].

In line with our study’s findings, the Department of Veteran Affairs HERC Health Economics Seminar, specifically Jason Hockenberry’s presentation on ‘The Cost of Readmissions: Implications for Reimbursement Policies,’ highlights the challenge of establishing a substantial relationship between hospital readmission rates and costs. Hockenberry’s observations indicate that the coefficient on the hospital readmission rate from the previous period is notably small and statistically insignificant. This aligns with the complex nature of the financial implications of readmissions, where even a negative cost effect of USD 12.00–USD 31.00 is deemed a minor impact (Department of Veteran Affairs HERC Health Economics Seminar, 2018).”

In line with other studies, our results indicated that among the three 30-day risk-standardized unplanned readmission measures reported, THA/TKA reported the highest attributable cost per readmission, USD 21,346.50 (95% CI, USD 20,818.14–21,871.85) [29,30,31,32]. These results align with the CMS adding THA/TKA to the HRRP because of the high prevalence, its increased number of readmissions, and the high overall Medicare expense for this measure [33].

The results of our estimates suggest questions on the unintended cost implications of the HRRP with the various five measures grouped together and individually. First, within our scope, this study is the first meta-analysis to simulate the cost of readmissions after the onset of the HRRP. Despite the lack of experimental studies, observed variations in the cost of readmission provide evidence that the CMSs were right to expand the HRRP measures. More financial performance studies are warranted to inform policy since the HRRP was designed to improve the quality by reducing hospital readmissions and decreasing CMS spending [34].

### Limitations

Our research encountered several limitations. Firstly, the inclusion of different pathological conditions in the studies created challenges in consolidating the data. Secondly, most studies lacked the mention of controls, specifically 30-day unadjusted mortality rates, which posed significant obstacles to conducting a meta-analysis. Thirdly, the cost estimates derived from peer-reviewed articles exhibited considerable heterogeneity. Authors presented various types of costs and financial performance measures, with discrepancies in cost reporting methods. Despite our efforts to mitigate these variations through a rigorous review process, we primarily focused on mean cost estimates for inclusion. Indeed, for certain readmission measures, we excluded some articles due to the extent of heterogeneity observed. To address the inherent uncertainty and heterogeneity in our data, we employed Monte Carlo simulations as part of our analysis. There is a clear need for robust studies to comprehensively assess the cost of readmissions.

There is the possibility that our results contain some underestimations resulting from the population under study. The study is limited to an adult population readmitted to acute care hospitals. We excluded non-acute, long-term care facilities, and pediatric acute hospitals. The total attributable costs for readmission for the entire US healthcare system are most likely higher, warranting an increase in readmission reduction initiatives. Finally, we acknowledge the presence of comorbidities that can impact the average readmission cost. Although we attempted to account for co-morbidities and primary diagnosis in our included studies, it is possible that this might not be a complete list. 

## 5. Conclusions

Our study offers valuable insights into the financial burden imposed by re-admissions on acute care hospitals, providing robust estimates of attributable cost resources for readmissions across various medical conditions. By quantifying the mean costs of 30-day readmissions for conditions such as AMI, HF, and THA/TKA, our findings shed light on the economic impact of readmissions and highlight areas where healthcare resources are being allocated.

One of the primary benefits of our study is its provision of concrete estimates that can inform decision-making and resource allocation strategies for healthcare stakeholders, including hospital administrators, policymakers, and payers. By understanding the financial implications of readmissions, stakeholders can develop targeted interventions and quality improvement initiatives to reduce readmission rates and optimize healthcare spending.

Furthermore, our study opens avenues for future research by identifying persistent trends in readmission costs and emphasizing the need for continued efforts to address this challenge. Future studies can build upon our findings by investigating the effectiveness of specific interventions and strategies to reduce readmissions and improve overall healthcare outcomes. Additionally, exploring the impact of demographic and clinical factors on readmission costs could provide further insights into the drivers of healthcare expenditure.

In light of the ongoing emphasis on value-based care and healthcare cost containment, our study underscores the importance of addressing readmissions as a critical component of healthcare quality improvement efforts. By addressing the financial implications of readmissions, hospitals can better align their resources and interventions to improve patient outcomes while optimizing healthcare spending.

## Data Availability

Data are contained within the article.

## Appendix A

**Table A1:** Results of systematic analysis.

Article	Author	Study Period/Sample/Design/Location	Independent Variables (IVs)	Dependent Variables	Data Source	Results
Costs associated with unplanned readmissions among patients with heart failure with and without hyponatremia	[35]	2014–2016/national sample of over 1000 hospitals/retrospective cohort	30-day readmission rate	Incremental cost	Premier healthcare, HCUP	Readmission cost when using HCUP was USD 547 and USD 569 for patients using premier database
Return-on-Investment (ROI) Analyses of an Inpatient Lay Health Worker Model on 30-Day Readmission Rates in a Rural Community Hospital	[36]	2010–2015/single hospital/cross-sectional	30-day readmission rate	Cost, return on investment	The hospital’s utilization and financial data	If the hospital was an ACO, as was the case for this study’s community hospital, the ROI significantly increased to USD 38.48 for every USD 1 spent on the BTH program
Measuring the Hospital Length of Stay/Readmission Cost Trade-Off Under a Bundled Payment Mechanism	[37]	1/2/08 and 31/12/08/acute care hospitals in New York/longitudinal	30-day readmission rate	Marginal cost	State inpatient databases (SID) and HCUP, AHA	The mean MC cost was USD 1857, and the mean MECR is USD 316; the mean difference between MC and MECR is USD 1541
The cost of hospital readmissions: evidence from the VA	[38]	2011/National/619,479 patients/cross-sectional	30-day readmission rate	Direct patient cost: fixed and variable	VA administrative sources	The variable cost among sub-groups was lower for readmission rates, ranging from USD 6077 for pneumonia to USD 8345 for heart attack
Sex-based differences in outcomes, 30-day readmissions, and costs following catheter ablation of atrial fibrillation: the United States Nationwide Readmissions Database 2010–14	[39]	2010–2014/National/54,597 admissions/retrospective cohort	30-day readmission rate	Cost	United States Agency for Healthcare Research and Quality (AHRQ) NRD	Among patients readmitted within 30 days, the median costs of readmission trended higher for females compared to males [USD 5774 (IQR USD 3286–10,661) vs. USD 5519 (IQR USD 3263–10,071); *p* = 0.076]
Outcomes, Costs, and 30-Day Readmissions After Catheter Ablation of Myocardial Infarct–Associated Ventricular Tachycardia in the Real World	[40]	2010–2015/4109 admissions/retrospective cohort/national	30-day readmission rate	Cost	HCUP state inpatient databases, the NRD	The median cost of readmission was USD 7932 (IQR, USD 4146–25,009)
Hospital Readmission and Costs of Total Knee Replacement Surgery in 2009 and 2014: Potential Implications for Healthcare Managers	[41]	2009–2014/30,000 patients/national sample/retrospective cohort	30-day readmission rate	Cost	HCUP state inpatient databases, the NRD	Costs per stay increased from USD 9929 to USD 11,904 over the four-year period, up a total of 20%
Risk Factors, Causes, and Costs of Hospital Readmission After Head and Neck Cancer Surgery	[42]	2010–2014/nationwide/retrospective cohort/9487patients	30-day readmission rate	Cost	HCUP state inpatient databases, the NRD	Mean cost per readmission was USD 15,916 standard error of the mean was USD 785; lower value was about USD 9000–USD 36,000
Causes, Risk Factors, and Costs of 30-Day Readmissions After Mitral Valve Repair and Replacement	[43]	2010–2014/national/retrospective cohort/76,342 patients	30-day readmission rate	Cost	HCUP state inpatient databases, the NRD	The mean cost for readmission was USD 15,397; lower value for readmission was USD 10,164 for arrythmia and USD 24,739 for infection
Nationwide Analysis of 30-Day Readmissions After Esophagectomy: Causes, Costs, and Risk Factors	[44]	2010–2014/national/retrospective cohort/76,342 patients	30-day readmission rate	Cost	HCUP state inpatient databases, the NRD	Median cost of readmission was USD 9660 (interquartile range, USD 5392 to USD 20,447)
Contribution of 30-day readmissions to the increasing costs of care for the diabetic foot	[45]	2012–2016/single hospital/prospective cohort/150 patients	30-day readmission rate	Cost	Primary data collection	The median hospital cost per admission was USD 20,111 (interquartile range, USD 12,589–33,254); attributable cost was cost USD 7.9 million over 4 years, of which USD 1.2 million (16%) was attributable to readmission costs
The Relative Importance of Post-Acute Care and Readmissions for Post-Discharge Spending	[46]	2007–2008/national/critical access hospitals/3217 patients/retrospective cohort	30-day readmission rate	Cost	Medicare fee-for service data	Average spending ranging from USD 1768 for MS-DRG 379 (GI hemorrhage) to USD 12,369 for MS-DRG 480 (hip and femur procedures); the interquartile range varied from USD 1245 for MS-DRG 192 (COPD) to USD 4393 for MS-DRG 281 and USD 7874 for MS-DRG 282 (both AMI)
Readmission after pancreatic resection: causes, costs and cost-effectiveness analysis of high versus low quality hospitals using the Nationwide Readmission Database	[47]	2010–2014/national/53,572 cases/retrospective cohort/	30-day readmission rate	Cost	HCUP national readmission databases, the NRD	The average cost of readmission was USD 15,563, the incremental adjusted cost of a major complication during the readmission was USD 38,028 ± 456
The Effects of Multiple Chronic Conditions on Adult Patient Readmissions and Hospital Finances: A Management Case Study	[48]	2010–2015/single hospital/retrospective cohort/2659	30-day readmission rate	Cost	Hospital data	Patients with 1 selected clinical condition present had the highest margin per admission (USD 2912); patients with 5 or more clinical conditions, on average, a total loss of USD 865 per admission
Relationship Between Hospital Financial Performance and Publicly Reported Outcomes	[49]	2008–2012/statewide/retrospective cohort/279 hospitals	30-day readmission rate	Revenue	Hospital annual financial data files from the Office of Statewide Health Planning and Development (OSHPD), CMS via the hospital comparison	Net revenue from operations from 2008 to 2012 (difference-in-differences estimates ranged from USD 8.61 to USD 6.77 million, *p* > 0.3 for all)
Costs And Clinical Factors Associated With 30- And 60-Day Hospital Readmission After Ventricular Tachycardia Ablation	[50]	2013/nationwide/cross-sectional/529 patients	30-day readmission rate	Cost (standard charge to cost calculations)	HCUP National Readmission Databases, the NRD	Costs for subsequent readmissions within 30- and 60 days post-ablation were USD 6973 and USD 7620
Treatment outcomes, 30-day readmission and healthcare resource utilization after pancreatoduodenectomy for pancreatic malignancies	[51]	2014/nationwide/cross-sectional/4445 patients	30-day readmission rate	Cost, charges	HCUP National Readmission Databases, the NRD	The number of hospital days associated with readmission was 5548, with an in-hospital economic burden of USD 12.9 million (costs) and USD 43.7 million (charges)
Impact of Bipolar Disorder on Readmission Rates and Costs After Coronary Artery Bypass Grafting	[52]	2010–2014/412,949/retrospective cohort/nationwide	30-day readmission rate	Cost	National Readmission Database	Bipolar diagnosis did not significantly impact total hospital costs or length of stay of the index visit
Incidence, Cost, and Risk Factors for Readmission After Coronary Artery Bypass Grafting	[53]	2013–2014/288,059/retrospective cohort/national	30-day readmission rate	Cost	2013 and 2014 Nationwide Readmissions Database (NRD)	Readmitted patients had a significantly cost (USD 49,528 USD 544.40 versus USD 41,014 USD 406.10) (all *p* < 0.001) compared with no readmissions
Inpatient costs, mortality and 30-day readmission in patients with central-line-associated bloodstream infections	[54]	2008–2010/398/single-hospital/prospective study	30-day readmission rate	Total cost and variable cost	Survey data	CLABSI was associated with c. USD 49,600 in excess total costs and USD 32,400 in excess variable costs
Readmission Rates and Their Impact on Hospital Financial Performance: A Study of Washington Hospitals	[25]	2012–2014/98 hospitals/retrospective cohort/statewide	30-day readmission rate	Operating revenues, operating expenses, operating margin	CMS hospital comparisons	The average operating revenues per patient is higher in 2014 than in 2013 by USD 9602 and in 2012 by USD 10,511; similarly, the mean operating expenses per patient is higher in 2014 than in 2013 by USD 9508 and in 2012 by USD 9436; the average operating margin in 2014 is higher by 2.56 percent points in 2013 and by 3.95 percentage points in 2012
Predictors and Costs of 30-Day Readmissions After Index Hospitalizations for Alcohol-Related Disorders in US Adults	[32]	2014/285,767 hospitalizations/cross-sectional/nationwide	30-day readmission rate	Cost	NRD	Index hospitalization costs were higher among readmitted patients (USD 8840 vs. USD 8036, *p* < 0.01)
Predictors of Cost and Incidence of 30-Day Readmissions Following Hospitalizations for Schizophrenia and Psychotic Disorders	[55]	2014/77,625 discharges/cross-sectional/nation-wide	30-day readmission rate	Cost	Nation-wide readmission database	The average index and readmission costs were USD 9285 and USD 8593, respectively
Does a reduction in readmissions result in net savings for most hospitals? an examination of Medicare’s Hospital Readmissions Reduction Program	[4]	2016/2465 hospitals/cross-sectional/Nation-wide	30-day readmission rate	Reimbursement gains	Hospital comparison dataset	For an average hospital, avoiding one excess readmission would result in reimbursement gains of USD 10,000–USD 58,000 for Medicare discharges
One-year costs of medical admissions with and without a 30-day readmission and enhanced risk adjustment	[56]	2000–2011/retrospective cohort/national/4684 hospitalizations	30-day readmission rate	One-year subsequent Medicare spending (USD)	MCBS cost and use files	The unadjusted subsequent one-year Medicare spending among those readmitted (USD 56,856) was 60% higher than that among the non-readmitted (USD 35,465)
Hospital readmission with Clostridium difficile infection as a secondary diagnosis is associated with worsened outcomes and greater revenue loss relative to principal diagnosis: a retrospective cohort study	[57]	2009–2013/retrospective cohort/4 states/5468 hospitalizations	30-day readmission rate	Cost	State Inpatient Databases (SID), a part of the Health Care Utilization Project (HCUP)	Adjusted 30-day readmission cost and risk was lower in PrCDI (OR = 0.84; 95% CI 0.80, 0.88) and SrCDI (OR = 0.97; 95% CI 0.94, 1.01) than non-CDI
Comparison of Causes and Associated Costs of 30-Day Readmission of Transcatheter Implantation Versus Surgical Aortic Valve Replacement in the United States (A National Readmission Database Study)	[58]	2013/retrospective cohort/national/5468 hospitalizations	30-day readmission rate	Cost	NAD, HCUP	The 30-day cumulative costs were higher for the 2 endovascular TAVI (USD 51,025 vs. USD 46,228; *p* = 0.03) and transapical TAVI (USD 59,575 vs. USD 45,792; *p* < 0.01)
The cost of preventing readmissions: why surgeons should lead the effort.	[59]	2012/single-hospital/cross-sectional/576	30-day readmission rate	Cost	United University Health Consortium	Calculated net profit for readmission was USD 144
Effects of an Acute Care for Elders Unit on Costs and 30-Day Readmissions	[60]	2010/single-hospital/cross-sectional	CMI	Cost	UAB hospital administrative database,	Adjusted cost ratios revealed significant cost savings for patients with low (0.82; 95% CI, 0.72–0.94) or moderate (0.74; 95% CI, 0.62–0.89) CMI scores; care was cost neutral for patients with high CMI scores (1.13; 95% CI, 0.93–1.37)
How Much Does a Readmission Cost the Bundle Following Primary Hip and Knee Arthroplasty?	[30]	Retrospective cohort	30-day readmission rate	Cost	CMS claims	Readmitted patients had an average 90-day episode-of-care cost of USD 42,923 compared to USD 18,514 for no readmitted patients (*p* < 0.001; Table A1);patients who were readmitted generated significantly higher subacute care costs (USD 5201 vs. USD 3707, *p* < 0.001), home health aide costs (USD 1796 vs. USD 808, *p* < 0.001), and overall post-acute care costs (USD 28,064 vs. USD 4021, (*p* < 0.001)
Incidence, Cost, and Risk Factors for Readmission After Coronary Artery Bypass Grafting	[53]	Retrospective cohort	30-day readmission rate	Cost	HCUP	Readmitted patients had a significantly higher cost (USD 49,528 USD 544.40 versus USD 41,014 USD 406.10) (*p* < 0.00)
Length of Stay and Cost of Pediatric Readmissions	[61]	Retrospective cohort	30-day readmission rate	Cost	administrative database that contains information on	Readmission cost USD 6328 (95% CI: USD 6184–6475), respectively
Institutional Cost of Unplanned 30-Day Readmission Following Open and Endovascular Surgery	[62]	Retrospective cohort	30-day readmission rate	Cost	The American College of Surgeons National Surgical Quality Improvement Program (ACS NSQIP)	The mean costs for 30-day unplanned readmission for open and endovascular procedures were USD 19,117 and USD 17,887, respectively (*p* = 0.635)
Frequency, Cost and Risk Factors of Readmissions among Severe Sepsis Survivors	[63]	Observational cohort	30-day readmission rate	Cost	HCUP	The mean cost of each readmission was USD 25,505 (standard deviation USD 38,765)
The Readmission Event after Vascular Surgery: Causes and Costs	[64]	Retrospective cohort	30-day readmission rate	Cost	Hospital data	The median hospital cost for readmission for wound complications was 29,723 USD (interquartile range 23,841–36,878), and for cardiac complications was 39,784 USD (26,305–46,918); the median cost of readmission for bypass graft occlusion was 33,366 USD (20,530–43,170)
Costs and Risk Factors for Hospital Readmission After Periprosthetic Knee Fractures in the United States	[65]	Retrospective cohort	30-day readmission rate	Cost	HCUP	ORIF cost USD 25,539 and revision THA cost USD 37,680, with associated readmissions costing 15,269 and 16,806, respectively
Proportion and Cost of Unplanned 30-Day Readmissions After Sepsis Compared with Other Medical Conditions	[66]	Retrospective cohort	30-day readmission rate	Index admissions, length of stay	CMS cost reports	The estimated mean cost per readmission was highest for sepsis compared with the other diagnoses (USD 10,070 [95% CI, USD 10,021–10,119] for sepsis, USD 8417 [95% CI, USD 8355–8480] for COPD, USD 9051 [95% CI, USD 8990–9113] for heart failure, USD 9424 [95% CI, USD 9279–9571] for AMI, and USD 9533 [95% CI, USD 9466–9600] for pneumonia; *p* < 0.005 for all pairwise comparisons)
Predictors and Cost of Readmission in Total Knee Arthroplasty	[31]	Retrospective cohort	30-day readmission rate	Cost	HCUP	The overall median cost for each readmission was USD 6753 ± 175
